# Towards equity in immunisation

**DOI:** 10.2807/1560-7917.ES.2019.24.2.1800204

**Published:** 2019-01-10

**Authors:** Tammy Boyce, Amelie Gudorf, Catharina de Kat, Mark Muscat, Robb Butler, Katrine Bach Habersaat

**Affiliations:** 1 Independent consultant, Cardiff, United Kingdom; 2 Independent consultant, Copenhagen, Denmark; 3 Vaccine-preventable Diseases and Immunization programme, World Health Organization Regional Office for Europe, Copenhagen, Denmark

**Keywords:** epidemiology, inequity, social determinants of health, policy, public health policy, vaccines, immunisation, Europe, outbreaks, school outbreaks

## Abstract

In the World Health Organization (WHO) European Region, differences in uptake rates of routine childhood immunisation persist within and among countries, with rates even falling in some areas. There has been a tendency among national programmes, policymakers and the media in recent years to attribute missed vaccinations to faltering demand or refusal among parents. However, evidence shows that the reasons for suboptimal coverage are multifactorial and include the social determinants of health. At the midpoint in the implementation of the European Vaccine Action Plan 2015–2020 (EVAP), national immunisation programmes should be aware that inequity may be a factor affecting their progress towards the EVAP immunisation targets. Social determinants of health, such as individual and household income and education, impact immunisation uptake as well as general health outcomes – even in high-income countries. One way to ensure optimal coverage is to make inequities in immunisation uptake visible by disaggregating immunisation coverage data and linking them with already available data sources of social determinants. This can serve as a starting point to identify and eliminate underlying structural causes of suboptimal uptake. The WHO Regional Office for Europe encourages countries to make the equitable delivery of vaccination a priority.

Despite the success of routine childhood immunisation programmes in reducing the incidence of vaccine-preventable diseases, immunisation uptake varies among countries, and among groups and districts within countries in the World Health Organization (WHO) European Region. There are also differences in coverage between the different scheduled vaccines. Inequity in uptake of routine vaccines has contributed to an accumulation of susceptible individuals in several countries of the Region [1,2] and hence also to the continued occurrence and spread of some vaccine-preventable diseases [3].

Inequities in health are associated with the social determinants of health, and inequities in immunisation are related to the concepts of social justice, fairness and ethics (Box 1)

Box 1. Concepts of equity and immunisation

**Inequity in immunisation:** Avoidable differences in immunisation coverage between population groups that arise because barriers to immunisation among disadvantaged groups are not addressed through policies, structures, governance or programme implementation [4,8].

**Equitable access to vaccines:** All individuals are offered the same vaccines through delivery services that are tailored to meet their needs.

**Social determinants of health:** The underlying conditions in which people are born, grow, live, work and age [27]. These determinants include parental income, education, living standards, gender equity, distribution of power, policy frameworks and social values.

## Commitment to equitable extension of vaccination services

In 2014, all 53 countries in the Region committed to achieve the six goals and five objectives of the European Vaccine Action Plan 2015–2020 (EVAP) [4]. Unfortunately, progress towards Objective 3, equitably extending the benefits of vaccination to all, and towards Goal 4, meeting regional vaccination coverage targets, has been slow [5]. The tendency among many national programmes, policymakers and the media in recent years has been to attribute decreasing or suboptimal vaccination uptake to parental concerns about vaccines or refusal, but this is only part of the problem. Evidence shows that the reasons for suboptimal coverage are multifactorial, and social determinants and systems-related barriers can play an equally or more important role, depending on the context [6,7]. Targeted studies with the beneficiaries are needed to understand which barriers are most critical to address. EVAP’s Objective 3 specifically states that “the benefits of vaccination are [to be] equitably extended to all people” [4], however, this key pathway which will help reach EVAP goals has not yet been sufficiently explored or used.

At the midpoint of EVAP, all national immunisation programmes should investigate the extent to which equity is an issue that affects their progress towards EVAP’s goals and targets (Box 2).

Box 2. Critical actions in addressing inequities in immunisation

• Acknowledge that immunisation coverage may be affected by social determinants and that parental concern about vaccination is only one of several potential reasons for suboptimal uptake;

• Reveal and monitor disaggregate data to reveal inequities in uptake (e.g. by income of parent, geographical region, age, ethnicity);

• Conduct research to identify root causes of identified inequities;

• Apply an equity focus in all immunisation-related activities by first considering how population groups may be impacted differently;

• Ensure fair and inclusive structures, policies and decision-making that goes beyond prioritisation based on cost-effectiveness.

## Identifying inequities in immunisation

Acknowledging that immunisation coverage may be affected by social determinants is an important step in addressing those differences in uptake that arise from inequity in vaccine delivery and access.

Correlating data on social determinants with vaccination coverage data can open a window to inequities influencing immunisation uptake, uncover a great potential for improvement of services and create an opportunity to increase vaccination coverage [8].

National immunisation uptake statistics do not usually provide sufficient detail to identify which local populations are not fully vaccinated. There is a clear need to move beyond measuring the difference between worst- and best-performing geographical areas and to accurately identify who or which groups are not being immunised and where. Most countries that have undertaken to identify inequities in immunisation have found them – most often related to social determinants such as parental socioeconomic status, number of years in education and/or ethnicity [9-11].

Research on different vaccines in various countries has shown that immunisation uptake is related to the same factors associated with other health inequities and social determinants of health, e.g. parental number of years in education and level of income [12-16]. The collection and analysis of disaggregate data at district level has proven useful to identify where inequities exist. For example in Wales, disaggregate data are routinely used to monitor socioeconomic inequalities in vaccination coverage in 4-year-old children and have also revealed that socioeconomic inequities in uptake are largest for vaccinations scheduled for older children [17,18]. In Ireland, disaggregate data analysis led to identifying a large socioeconomic gradient in infant vaccination, a problem previously unknown and not addressed [19]. A range of similar studies exist, bearing witness to the correlation between vaccination coverage and social determinants and demonstrating the need for more countries to use similar methods to identify inequities in uptake [20-23].

## From data to action

Treating all people the same will not necessarily reduce inequities in immunisation. There is no single way to ‘start’ to address inequities in immunisation, in some countries it may be necessary to develop policies, in others to adapt services, in others to develop systems to analyse and disaggregate data and in other countries to maintain and improve these disaggregate data. Addressing inequities is not a one-off action, it is a shift in conceptualising how services are delivered and how the goals and targets are set.

The first step in understanding inequities in immunisation is making inequities visible [20,21]. Understanding *who* is not immunised will help to understand *why* they are not immunised. Good quality, robust disaggregate data should be able to identify, map and track populations affected by inequities [22]. The goal should be for each country to analyse immunisation uptake data to identify presence or absence of inequities. This requires immunisation uptake data to be disaggregated by key determinants of inequalities: (i) socioeconomic status, (ii) geographical location, (iii) educational status of parents and (iv) ethnicity and migration status.

Once pockets of un- or under-vaccination in specific geographic areas or among certain population groups are identified, national programmes can research the barriers that prevent some individuals from getting vaccinated (for example, barriers related to individual beliefs, attitudes and knowledge as well as those related to access, cost and service provision) and identify interventions to address them. Identifying underlying structural causes allows countries to design equitable immunisation services, remove barriers to immunisation and ensure that the benefits of immunisation reach every child [1,17,23-26].

Immunisation services alone cannot address the social determinants of health. However, immunisation programmes should consider these factors and adapt vaccine service delivery to meet the needs of all populations to increase uptake. If not seen and designed through an equity lens, immunisation programme activities can in fact increase inequity [27]. There is a growing body of research, including systematic reviews, showing that multi-component, locally designed interventions are most effective in reducing inequities in immunisation uptake [15,28]. Inequities are not resolved by providing the same immunisation services to all; they are resolved by providing different immunisation services that satisfy the needs of all. Flexible and opportunistic immunisation programmes and good relationships between healthcare services and parents appear to improve vaccination coverage and reduce inequities [29]. Flexible interventions and services involve considering where immunisations are delivered and who administers vaccines, as well as providing multiple offers of immunisation.

### Where immunisations are delivered

Equitable immunisation programmes consider where it is easiest for families and individuals to be vaccinated. Vaccines can be delivered outside of health clinics, for instance in schools, pharmacies, community centres, hospitals or at home. For example, Belgium offered school-based vaccination against human papillomavirus (HPV), which increased rates of vaccination initiation/completion and lowered inequalities based on socioeconomic factors [30].

### Who administers vaccines

In some countries in the WHO European Region, only licensed family doctors are able to vaccinate. This may limit the flexibility of a service and add unnecessary costs. Enabling other healthcare workers such as nurses, midwives, school nurses and pharmacists to vaccinate may help increase equity. For example in the UK, school nurses’ familiarity with their students and their established relationships with socially excluded communities were key to increasing uptake among girls who did not attend or who missed doses of the HPV vaccine [31].

### Multiple offers of immunisation

The WHO Missed Opportunities for Vaccination strategy recommends any child or adult eligible for vaccination coming to a health service (for whatever reason) should be offered needed vaccines during their visit. This means offering vaccinations during visits to health services for curative services (e.g. treatment of fever, cough, injuries) or preventive services (e.g. parental classes), as well as offering them to accompanying family members [32]. For example, Scotland addressed inequities in their immunisation programme by offering vaccines many times and found it was “effective in minimising socioeconomic variation in the uptake of routine HPV immunisation in girls”. [33]

In the WHO European Region, some countries have mandatory vaccination policies, however, it is yet to be studied when and how such policies reduce inequities in immunisation uptake. Whether a country chooses to mandate vaccination or not, all 53 Member States of the Region have agreed to a set of immunisation goals in the European Vaccine Action Plan. It is up to the national health authorities to take measures suitable to their national context and ensure equitable and high immunisation coverage hereby protecting their citizens from life-threatening diseases.

## The wider benefits of improving equity in immunisation uptake

Equitable immunisation policies, like all equitable health policies, generate wider health, social, political and economic benefits [34]. Immunisation is a powerful method to attract people into healthcare, especially the most vulnerable [35]. Improving equity in immunisation can therefore also improve coverage of other health interventions [6]. 

EVAP suggests that countries in the Region ensure that every individual is eligible to receive all appropriate vaccines, irrespective of their geographic location, age, gender, educational level, socioeconomic status, ethnicity, nationality or religious or philosophical affiliation [3]. Governments are tasked with creating fair and inclusive structures and policies, in partnership with immunisation teams, health professionals and the recipients of vaccines, all working together to reduce inequities in health and in vaccination uptake. To support this work, organisations such as the WHO Regional Office for Europe works continuously to share evidence and normative guidance and to help countries learn from each other‘s work through the Tailored Immunization Programmes (TIP) [36]. The TIP helps countries identify the root causes of under-vaccination. 
